# Strong dependence of a pioneer shrub on seed dispersal services provided by an endemic endangered lizard in a Mediterranean island ecosystem

**DOI:** 10.1371/journal.pone.0183072

**Published:** 2017-08-21

**Authors:** Constanza Neghme, Luís Santamaría, María Calviño-Cancela

**Affiliations:** 1 Department of Biology, University of La Serena, La Serena, Chile; 2 Doñana Biological Station (EBD-CSIC). C/ Américo Vespucio, s/n, Isla de la Cartuja, Sevilla, Spain; 3 Evolutionary Ecology and Conservation Lab., Department of Ecology and Animal Biology, School of Sciences, Campus Lagoas-Marcosende, Vigo, Spain; Universidade da Coruna, SPAIN

## Abstract

The accelerating rate of vertebrate extinctions and population declines threatens to disrupt important ecological interactions, altering key ecosystem processes such as animal seed dispersal. The study of highly specialized mutualistic interactions is crucial to predict the consequences of population declines and extinctions. Islands offer unique opportunities to study highly specialized interactions, as they often have naturally depauperated faunas and are experiencing high rates of human-driven extinctions. In this study, we assess the effect of seed dispersal on seedling recruitment of *Ephedra fragilis* (Ephedraceae) on a Mediterranean island ecosystem. We used field data and stochastic simulation modeling to estimate seed fate and recruitment patterns of this pioneer shrub typical of arid and semiarid areas, and to estimate the dependence of recruitment on the lizard *Podarcis lilfordi* (Lacertidae), its only known seed disperser. *Ephedra fragilis* recruitment highly depended on lizards: lizards produced 3.8 times more newly-emerged seedlings than non-dispersed seeds and no seedlings from undispersed seeds survived the study period. Seed dispersal by lizards was mostly to open sites, which was key for the increased success observed, while undispersed seeds, falling under mother plants, suffered higher predation and lower seedling emergence and survival. The ability of this pioneer shrub to get established in open ground is crucial for vegetation colonization and restoration, especially on degraded lands affected by desertification, where they act as nurse plants for other species. Lizards are key in this process, which has important consequences for community structure and ecosystem functioning.

## Introduction

The accelerating rate of wildlife extinctions and population declines threatens to disrupt important ecological interactions, altering key ecosystem processes [1]. Plant recruitment is a crucial process that, in many plant species, depends on the services provided by animals for seed dispersal [2]. The mutualistic interaction between animal seed dispersers and fleshy-fruited plants has important consequences for plant demography and community structure [3,4]. In diverse communities, seed dispersers typically feed on the fruits of many different plant species, and seeds of a particular plant species are usually dispersed by many different animals. This makes these interactions diffuse, with low interdependence between plant and animal partners, which reduce the vulnerability of the community to co-extinctions and cascade effects (e.g. [3]). In addition, this diversity increases the probability of functional redundancy (i.e. several species having the same or similar roles and providing equivalent services). Functional redundancy attenuates the impact of the extinction or severe decline of particular species, which can be replaced in their role by their functional equivalents [5].

Nevertheless, in communities with low diversity, the number of potential interacting partners decreases, increasing the interdependence between them and reducing the probability of lost species being replaced by functional equivalents [6]. Islands often have naturally depauperated faunas, due to their isolation and small sizes, and are experiencing higher rates of human-driven extinctions and population declines than mainland areas, related mostly to habitat loss, biological invasions and climate change (e.g. [7]). By virtue of their biological simplicity, islands offer unique opportunities to study highly specialized mutualistic interactions and to determine the importance of key endemic species for ecosystem functioning [8]. This knowledge is crucial to predict the consequences of population declines and extinctions.

Island mutualistic interaction systems are also characterized by the importance of unusual animal partners. For instance, seed dispersal by lizards (saurochory) has been suggested to be chiefly an island phenomenon [9]. However, there are still few studies examining the degree of specialization of lizard-plant interactions and of dependence of plant species on the services provided by lizards in island ecosystems [10].

The quality of the seed dispersal service provided by an animal to a plant is determined by the recruitment success of the seeds it disperses [11]. This quality depends on the treatment seeds receive in the digestive tract and on where seeds are deposited by animals among sites with different suitability for recruitment. Thus, dispersal quality ultimately depends on the disperser physiology and behavior, in interaction with habitat and landscape features [12] and on the environmental requirements of the dispersed plant for successful germination, establishment and growth [13]. For instance, the microhabitats where seeds are dispersed (e.g. under shrubs or in open interspaces) might differ in light availability, temperature or humidity, which influence germination, emergence and seedling survival [14,15]. On the other hand, biotic factors are also determinants of plant recruitment, and for instance post-dispersal seed predation by vertebrates is one of the most critical biotic filter that decreases potential plant recruitment [16]. In addition, through animal dispersal, seeds escape from the high mortality under mother plants, where the high density of seeds attracts seed predators [17].

The main goal of this study is to assess the effect of seed dispersal by the highly frugivorous lizard *P*. *lilfordi* on recruitment of *Ephedra fragilis* Desf. (Ephedraceae), a pioneer evergreen gymnosperm shrub that produces succulent cones, which functionally resemble angiosperm fleshy fruits. To do that, we analyze the effect of seed dispersal on plant recruitment by studying the fate of dispersed vs. non-dispersed seeds throughout the subsequent stages of the recruitment process (i.e., arrival to different microhabitats, seed predation, and seedling emergence and survival), in order to determine the dependence of recruitment on animal dispersers.

## Methods

### Study species and study area

The study focused on *E*. *fragilis*, a dioecious evergreen gymnosperm shrub distributed in the Western Mediterranean and Macaronesian regions that usually inhabits arid and semiarid sclerophyllous shrublands in coastal areas. Ephedra is considered the closest living group of plants related to angiosperms [18]. *Ephedra fragilis* has cones (pseudo-flowers) and pseudo-fruits that functionally resemble angiosperm nectar-producing flowers and fleshy fruits, respectively. Pseudo-flowers produce pollination drops, enriched with sugar (see [19]), and are pollinated by wind and animals, with those open to animals reaching higher pseudo-fruit set and seed weight [20]. In Dragonera islet, the lizard *P*. *lilfordi* and insects (mainly Diptera) are the main visitors to pseudo-flowers, with lizards being the most frequent visitors (c. two times as many visits as insects; [20]). Three different seed dispersal syndromes have been described for this genus: one, considered the ancestral type, with succulent and brightly-colored cone bracts that is dispersed by frugivorous animals, other with dry, winged cone bracts dispersed by wind and a third syndrome with small, dry cone bracts and large seeds dispersed by seed-caching rodents [21]. *Ephedra fragilis* belongs to the first type, with females producing succulent cones, red or yellow in colour. Like other Ephedras, *E*. *fragilis* is a mast seeder [22], so that cone production is synchronized locally, which results in years of massive cone production. Fleshy cones are single-seeded (seeds c. 5 mm long and 2 mm wide), are available in summer (July, August and September) and are consumed by frugivorous animals, with *P*. *lilfordi* as the only known cone consumer and seed disperser in Dragonera islet (see also [23]). *Ephedra fragilis* seeds show no dormancy [24] and it can also reproduce vegetatively [25].

The study was conducted in Sa Dragonera Natural Park (39° 35′ N, 2° 19′E, 288 ha), an uninhabited islet off the NW coast of Mallorca. Mean annual rainfall is 350 mm and average annual temperatures are 17–18°C. July and August are the hottest months, with average maximum temperatures of 29–30°C, while January and February are the coldest, with average minimum temperatures of 8–8.5°C (Spanish Agency of Meteorology, www.aemet.es). The study was carried out at the northeastern tip of the islet (centered at 39°35′42′′N, 2°20′03′′W), where *E*. *fragilis* is abundant along a south to southeast-facing slope crossed by a path running in SW-NE direction that separates the two zones in which we conducted the study, uphill and downhill the path (“Zone A” and “Zone B” hereafter). The vegetation corresponds to a sclerophyllous shrubland (<2 m high) dominated by *Pistacia lentiscus* and *E*. *fragilis* (27.0% and 17.5% respectively), with presence of *Phillyrea angustifolia* (3.1%) and *Cneorum tricoccon* (3.7%), and with open ground (with no vegetation) covering 45.1% of the surface (percentage covers as measured in 150 m of transects across the study site; see S1 Appendix).

Regarding the fauna, the endemic Balearic lizard, *P*. *lilfordi*, stands out for its abundance (density 730 lizards/ha, estimated total population size in Dragonera c. 195.000 individuals [26]), which has been attributed to the low levels of predation. It is an omnivorous lizard, feeding mainly on insects, plant material and carrion [27], and is known to be an important pollinator and seed disperser [28,29,30]. Home ranges of *P*. *lilfordi* are *c*. 2–10 x 10^3^ m^3^, and average seed dispersal distances reach c. 70 m [23]. Known or potential predators of *P*. *lilfordi* in Dragonera include raptors, especially kestrels (*Falco tinnunculus*) and falcons (*Falco peregrinus*, *F*. *eleonorae*), seagulls, that capture lizards occasionally (mainly *Larus michaellis* and *L*. *audouinii*), and ship rats (*Rattus rattus*), which could predate on lizards [31]. *Rattus rattus* is very abundant in the islet and is also an important seed predator [32]. Fringilid passerines such as *Carduelis chloris* and *Fringila coelebs* could also predate on seeds. Regarding frugivores, *Sylvia atricapilla* is probably the most important frugivorous bird in the islet [33], but do not breed in Dragonera and is not present in summer [34], when the fruits of *E*. *fragilis* are available. *Sylvia melanocephala* and the endemic *S*. *sarda balearica*, although less frugivorous [33], are present in the islet in summer (estimated population size of 251 to 500 pairs and 101 to 250 pairs, minimum and maximum estimates in each case, respectively; [34]) and could occasionally consume some fruits, as well as *Turdus merula* (11 to 50 pairs; [34]). Due to the reduced number of frugivorous birds in summer, in contrast with the superabundant lizards, the role of passerine birds seems negligible. In addition, a preliminary survey, conducted in 150 m of transects to determine the main microhabitats of seed arrival through animal dispersal, revealed that all faeces with *E*. *fragilis* seeds (a total of 140) were of lizards (no bird faeces with *E*. *fragilis* seeds were found). Moreover, we never observed *E*. *fragilis* fruit consumption by birds and never found any bird faeces with *E*. *fragilis* seeds in the island, which lead us to conclude that *P*. *lilfordi* seems the only seed disperser of *E*. *fragilis* (see also [23]).

We selected the three most important microhabitats for the study, in which we analyzed the fate of seeds in the consecutive stages of the recruitment process, taking into account the percentage cover and the distribution of lizard faeces (see S1 Appendix). These were open ground, i.e. areas not covered by vegetation (Open hereafter), *E*. *fragilis* (Ephedra hereafter) and *P*. *lentiscus* (Pistacia hereafter).

### Microhabitat use by lizards

**Fig 1:**
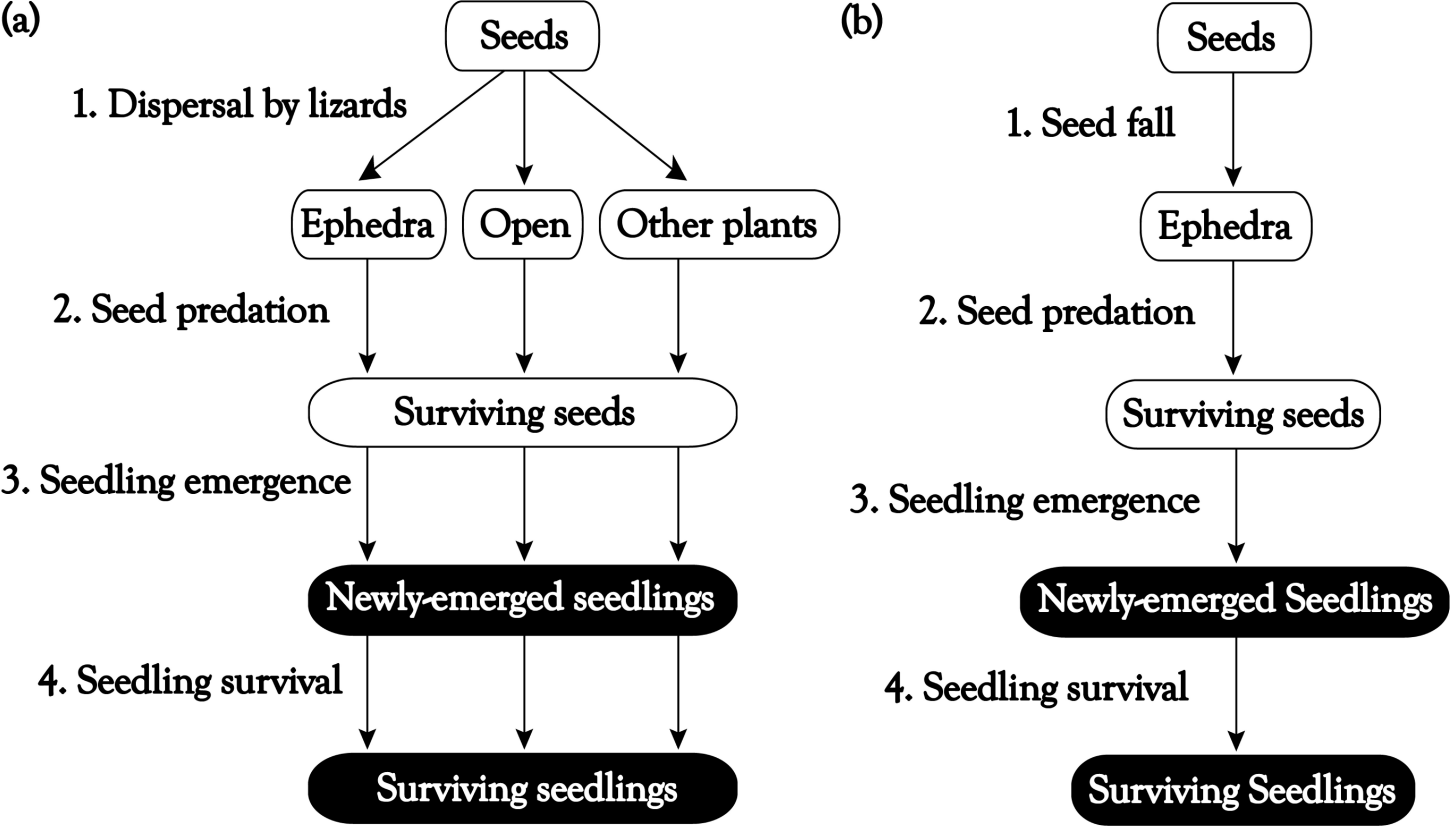
Model flow diagrams. Flow diagrams of the model for (a) seeds dispersed by lizards and (b) undispersed seeds, with recruitment stages (in ovals) through seedling recruitment and transitions between stages (1–4).

We estimated the use of microhabitats made by Balearic lizards, *P*. *lilfordi*, the only known seed disperser of *E*. *fragilis* in the study area (see “Study species and study area” section). In order to estimate the use of microhabitats by lizards we carried out direct observations along four 50 m transects running in SW–NE direction approximately parallel to the path that cross the study area, two transects in Zone A and two in Zone B, at a distance of c. 25 m and 50 m from the path, respectively. Observations were made in July 2007 in 5 separated days and by the same observer, walking slowly (average pace of 470 m per hour) and recording, for all lizards observed within a maximum of 7 m from the transect, the type of microhabitat in which they were spotted. We used these probabilities to parameterize the stochastic model in the seed dispersal stage (Stage 1. Dispersal by lizards in Fig 1A), since the proportion of time spend by lizards in each microhabitat determines the probability of seed dispersal to those microhabitats [23].

### Seed predation

In early August 2007 we exposed seeds to predation in a total of 60 trays, with 10 seeds each, placed in the three main microhabitats of seed arrival and seedling emergence, i.e. Open, Ephedra and Pistacia, in the two study zones (A and B), with 10 trays per microhabitat in each zone. Trays were grouped in sets of one replicate for each microhabitat (one in open, one in Ephedra and one in Pistacia) that were separated a maximum of 4 m from each other, while different sets (a total of ten) were separated at least 10 m from each other. Trays were made out of plastic trellis, 10 cm x 10 cm wide and 3 cm high, open on top so that seeds were available to all possible predators (e.g. rodents or granivorous birds). Trays were placed in the field in early August and were monitored for seed removal every 10 to 18 days afterwards for three months. Seven trays were destroyed or removed (1 in Open in Zone A, and 4 in Open and 2 in Ephedra in Zone B), probably due to disturbance by rats or rabbits, and were removed from the analyses. We used the probabilities of seed survival to predation to parameterize the stochastic model in the seed predation stage (Stage 2. Seed predation in Fig 1A and 1B).

### Seedling emergence and survival

In early winter (December 2007) we sowed in the field *E*. *fragilis* seeds collected from faeces (600) and from plants (600 seeds from yellow-fruited plants and 600 from red-fruited plants) in the study area. For seeds collected from plants we took 30 seeds from each of 20 plants of each morph (30 from each of 20 yellow-fruited plants and 30 from each of 20 red-fruited plants) but seeds from different individual plants were mixed within each treatment, to make a total of 600 seeds from each morph (yellow and red). Ten seeds from each treatment (a total of 30 seeds) were sown in each of 60 sowing stations, placed in the same microhabitats,zones and sets as described previously (10 in Open, 10 in Ephedra and 10 in Pistacia, in zones A and B, respectively). In each seed sowing station, we nailed to the ground a 20 x 20 cm metal mesh with 100 4 cm^2^ squares (2 cm x 2 cm). We selected randomly the location of the 30 seeds (10 per treatment) in the central 30 (6 x 5) cells of the grid. For each plot we had a map with the position of each sowed seed, in order to identify the treatment of each seed and of each of the seedlings that emerged. Seeds were sowed directly in the soil. Sowing stations were protected by a steel mesh (20 cm x 20 cm wide and 5 cm high; 2 cm hole size), that was nailed to the ground. Seed sowing stations were monitored for seedling emergence and survival every 10 to 30 days for a total of 200 days. We used the probabilities of seedling emergence and survival to parameterize the stochastic model in these stages (Stages 3 and 4 in Fig 1A and 1B).

### Statistical analyses

We fitted Generalized Linear Mixed Models with Binomial error distributions and logit link functions to the data on seed predation, seedling emergence and survival. We used the effects of microhabitat as a fixed factor on seed predation, and the effect of microhabitat and seed treatment (seeds taken from fruits collected in mother plants -yellow or red in colour- or from lizard faeces) as fixed factors on seedling emergence and survival (including interaction terms). The zone and the set were introduced as random factors in all cases. We used R [35] and lme4 to perform the analyses. Multiple pairwise comparisons of means were corrected with the sequential Bonferroni method [36].

### Estimation of the contribution of dispersers to plant recruitment using stochastic simulation

We estimated the percentage of seeds that successfully emerge and survive by means of stochastic simulations. In contrast to deterministic models, stochastic simulations allow us to take into account the stochastic nature of environmental conditions, thus better reflecting the complexity of the recruitment process. Our model is an adaptation of the model used in [37] and consists of a series of life stages (dispersed seeds, surviving seeds, newly recruited seedlings and surviving seedlings) connected by a series of processes (seed dispersal, post-dispersal seed predation, seedling emergence and seedling survival), each with its own set of empirical transition probabilities measured in the field, as described in previous sections, for both seeds dispersed by lizards and those that remain non dispersed (see Fig 1A and 1B). The original set of transition probabilities for each process obtained in the field (i.e. all the replicates used) was resampled 500 times by random selection with replacement [38] and the final output of each simulation iteration was the result of the product of the randomly selected transition probabilities at each stage (the probability of being dispersed to each microhabitat × the probability of surviving predation × the probability of seedling emergence × the probability of seedling survival). Thus, we obtained the percentage of seeds that become newly-emerged or surviving seedlings for those dispersed by lizards and those remaining undispersed. In order to estimate the probability of recruitment for seeds dispersed by lizards in each area, the probability obtained for each microhabitat was weighted by the relative cover of each microhabitat.

## Results

### Microhabitat use and seed dispersal by lizards

**Fig 2:**
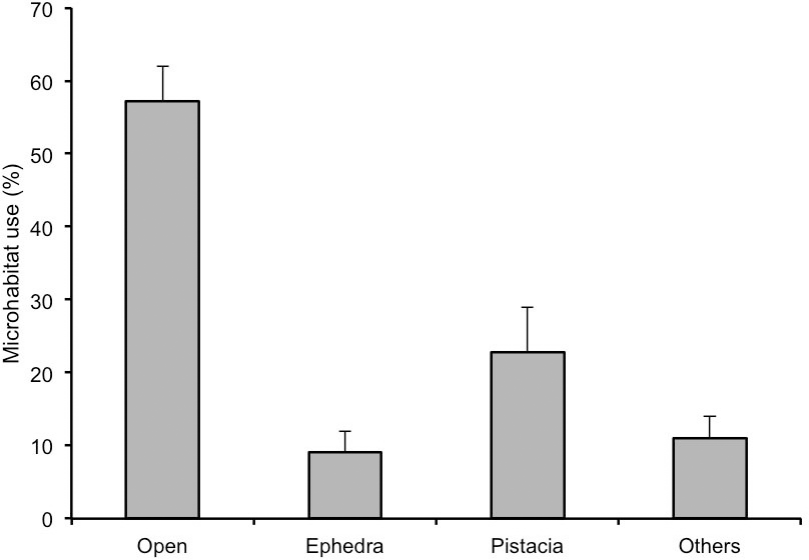
Microhabitat use by lizards. Percentage of lizard observations in different microhabitats (mean ± SE).

The open microhabitat was the most frequented by lizards, followed by Pistacia, Other plants and Ephedra (Fig 2). Open is thus the microhabitat where more lizard faeces containing *E*. *fragilis* seeds were found, with a small percentage arriving under Ephedra or Pistacia (S1 Appendix). Lizards showed a positive selection of open areas for depositing their droppings, with the percentage of droppings arriving to this microhabitat being higher than that expected by a random distribution among microhabitats (S1 Appendix). In contrast, Ephedra and Pistacia shrubs were negatively selected for dropping deposition.

### Seed predation

**Fig 3:**
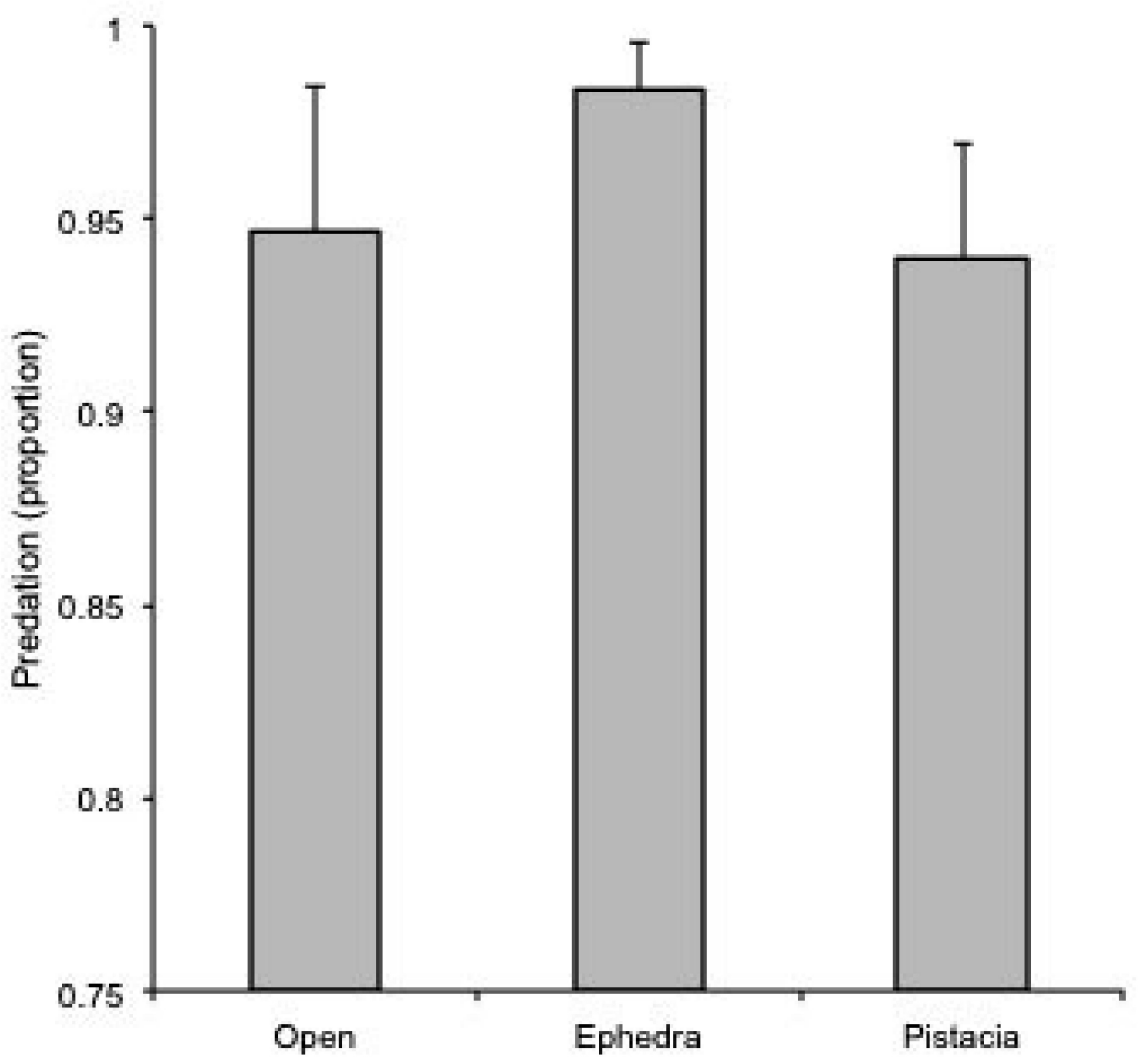
Seed predation. Proportion of seeds predated (mean ± SE) in each microhabitat.

There was a significant effect of the microhabitat on seed predation (*χ*^2^_2 d.f._ = 6.568, *P* = 0.038). Seeds beneath Ephedra had more predation than seeds in the open and beneath Pistacia (Fig 3).

### Seedling emergence and survival

**Fig 4:**
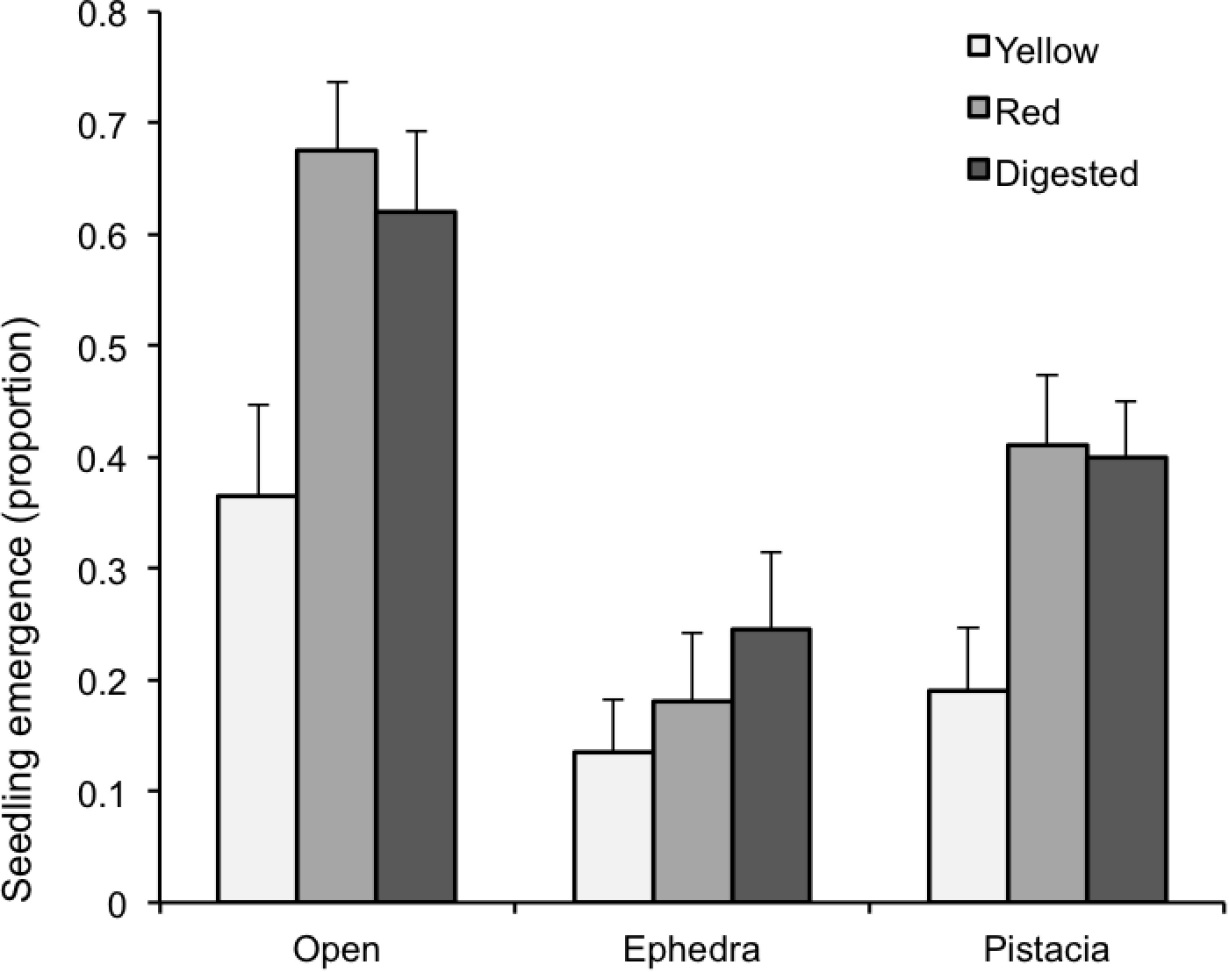
Seedling emergence. Proportion of seeds that emerged as seedlings after 200 days of monitoring in the different microhabitats and seed treatments (mean ± SE).

The microhabitat had also a significant effect in relation to seedling emergence (Table 1), with seeds beneath Ephedra showing lower germination probability than those beneath Pistacia or in the open, which had the highest, a pattern that was consistent in the three seed treatments (yellow and red morphs and digested seeds) (Fig 4). The seed treatment had also a significant effect on seedling emergence (Table 1). Non-digested seeds from yellow fruits had the lowest germination probability, while non-digested seeds from red fruits and seeds taken from lizard pellets showed similar probabilities (Fig 4).

**Table 1:** Seedling emergence and survival. Results of the analysis of the differences in seedling emergence and survival using a GLMM with binomial error distribution and logit link function.

		Seedling emergence		Seedling survival	
Factor	d.f.	Deviance (χ^2^)	*P* value	Deviance (χ^2^)	*P* value
Microhabitat	2	202.6	<0.001	57.2	<0.001
Treatment	2	77.2	<0.001	3.0	0.221
Microhabitat: Treatment	4	8.5	0.076	1.3	0.866

**Fig 5:**
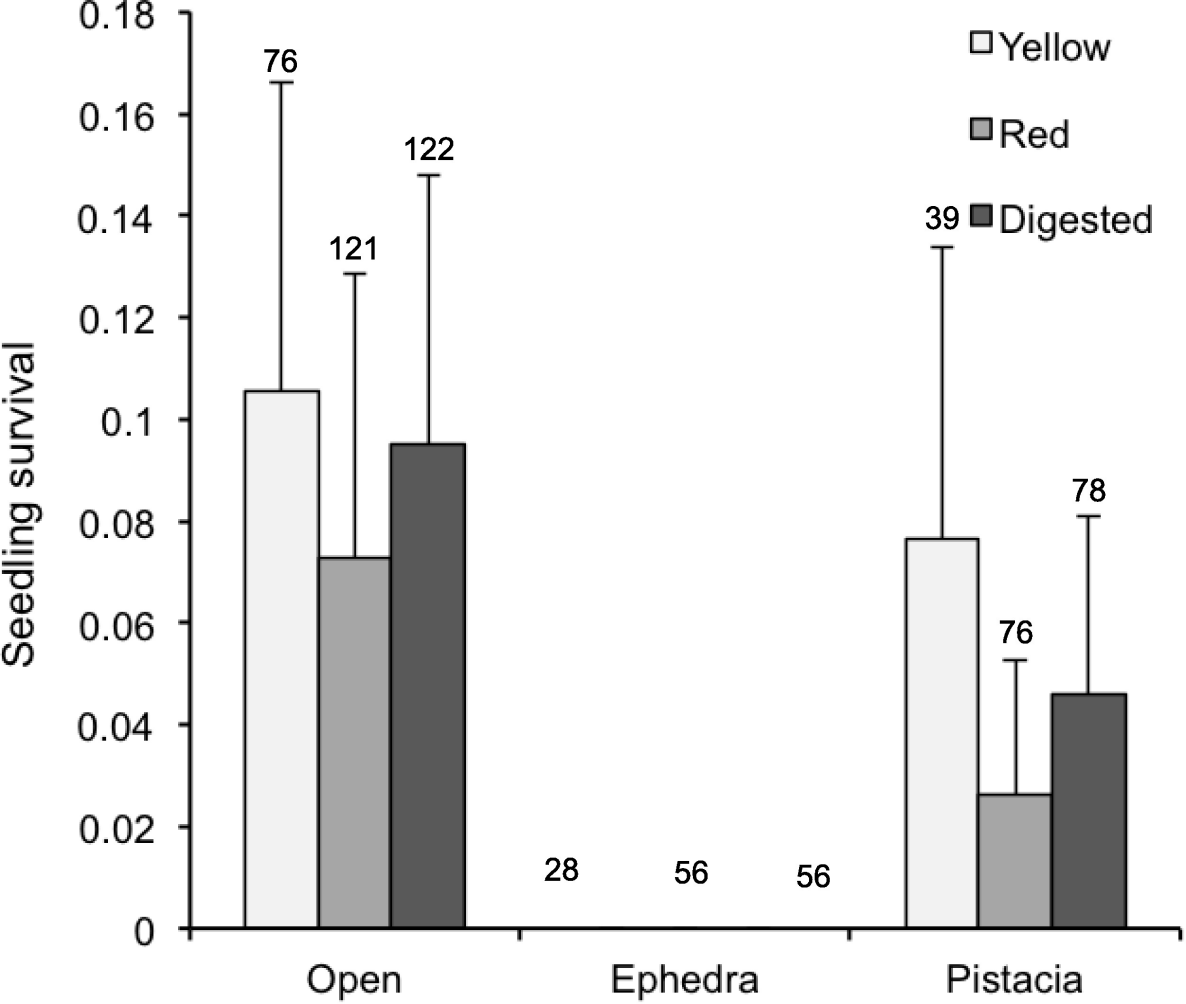
Seedling survival. Proportion of seedlings surviving in the studied microhabitats for seeds taken from yellow and red fruits and from lizard faeces (mean ± SE). Numbers on top of bars show the sample sizes in each case (i.e. number of emerged seedlings).

In regard to seedling survival, the microhabitat had also an important effect, with Ephedra being the only microhabitat where no seedling survival was observed (Fig 5). The effect of seed treatment (seeds from yellow or red fruits or from lizard droppings) was not significant, as was the interaction between treatment and microhabitat (Table 1).

### Estimation of the contribution of dispersers to plant recruitment using stochastic simulation

**Fig 6:**
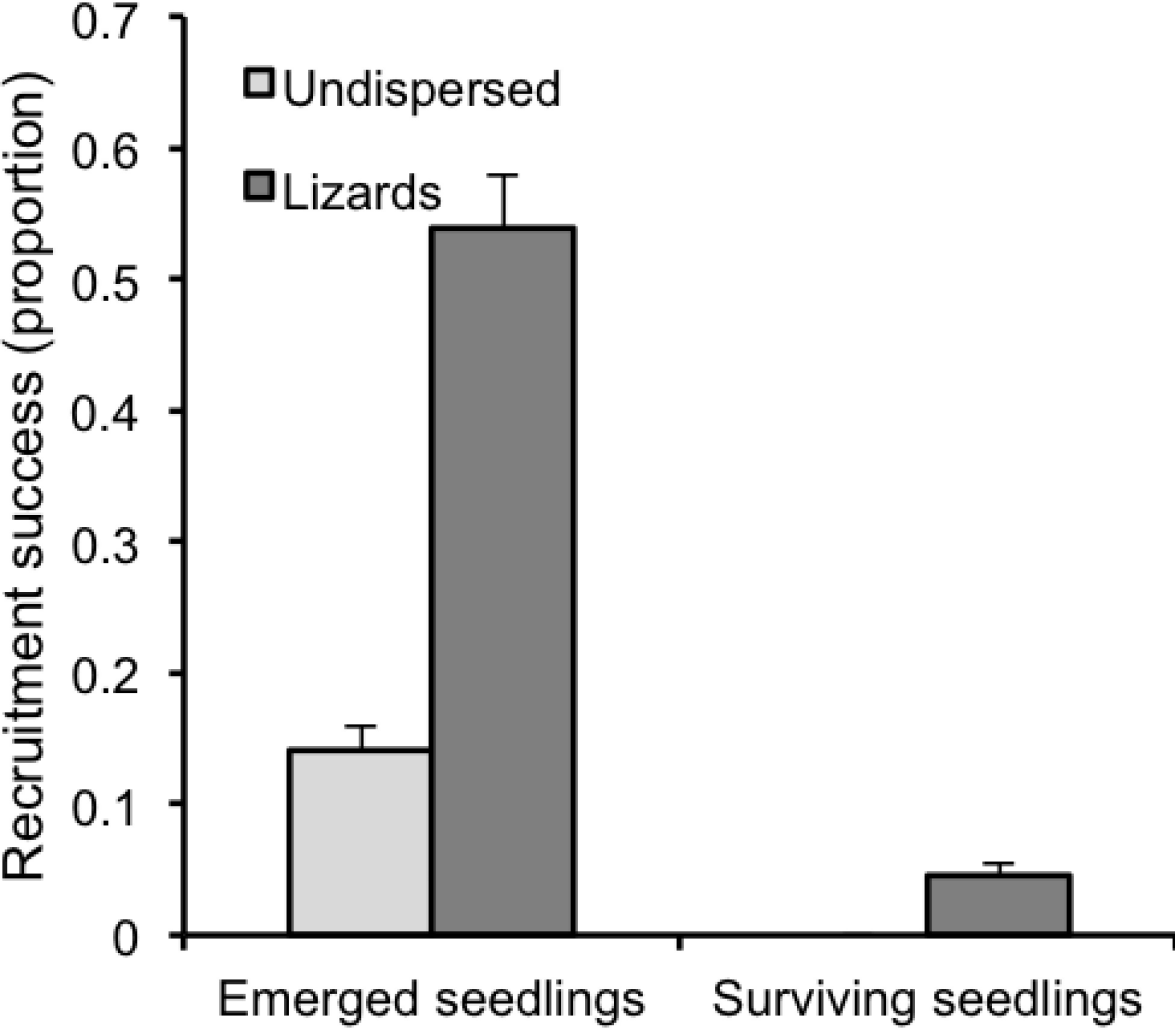
Seedling recruitment probability in seeds dispersed by lizards and undispersed seeds. Proportion of seeds of *Ephedra fragilis* that emerge as seedlings and survive (means ± SE), as estimated by the stochastic simulations.

According to our recruitment model, which integrates all the stages analysed before, dispersal by lizards increased *E*. *fragilis* recruitment by producing on average 3.8 times more newly recruited seedlings than non-dispersed seeds (Fig 6). The high seedling mortality in the first year was especially severe for seedlings emerging from non-dispersed seeds, with no survival being recorded (Fig 6). For seedlings emerging from dispersed seeds, mortalities reached on average 95.4% (Fig 6).

**Fig 7:**
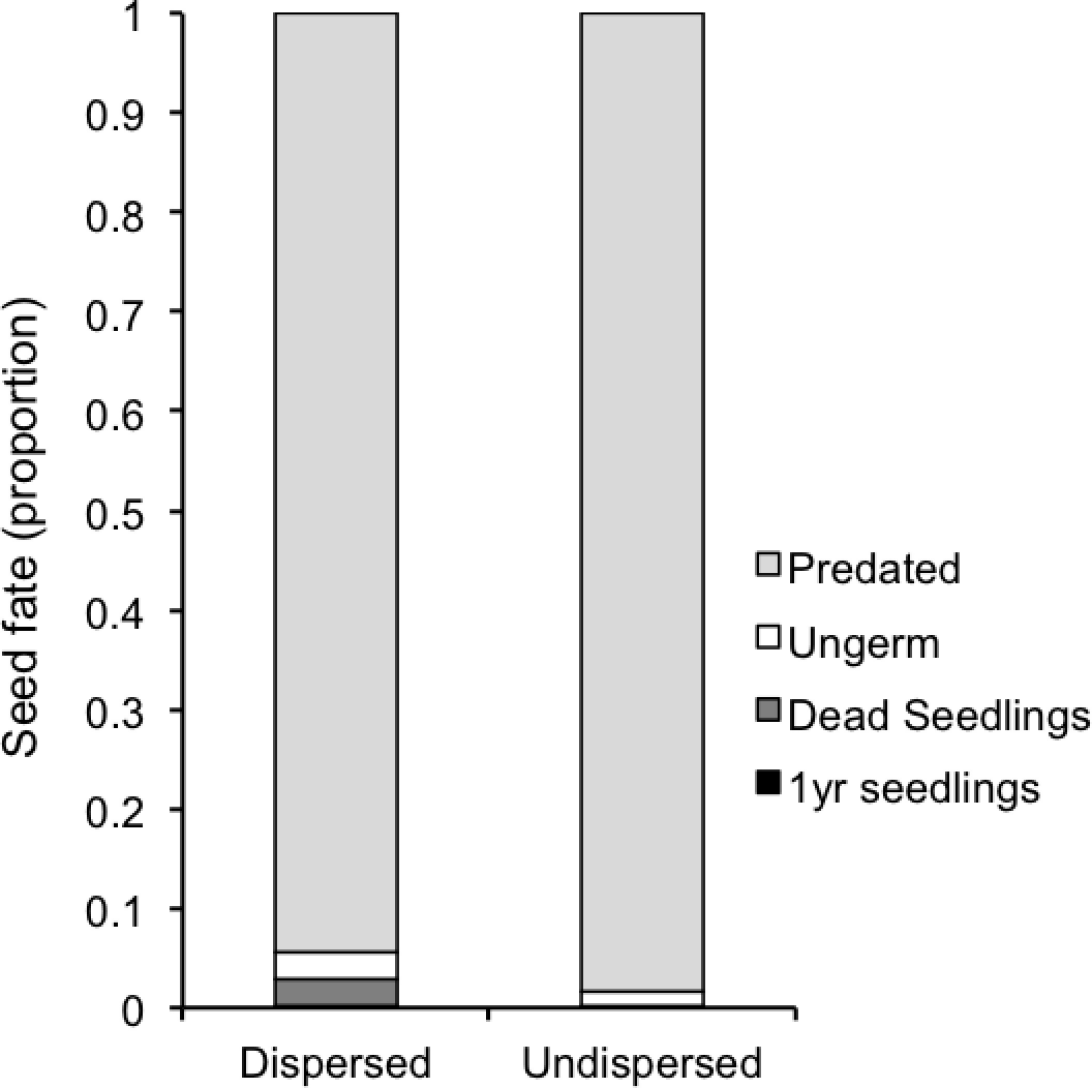
Seed fate. Fate of seeds dispersed by lizards and undispersed seeds. Bars show the mean proportion of seeds that are predated, those that remain ungerminated, those that emerge as seedlings, and of seedlings that survive.

Looking at the relative losses produced in each of the recruitment stages (Fig 7), seed predation stands out as the most hazardous stage, with higher mortality in undispersed seeds (98.3%) than in seeds dispersed by lizards (94.3%). For the seeds that remain alive, again the proportion of them that fail to emerge as seedlings was higher for undispersed seeds (85.4% on average in the two zones, respectively) than for lizard-dispersed seeds (47.7%). The seedling survival phase was also more hazardous for undispersed seeds, with no survival, in contrast with a mortality of 91.5% on average of the emerged seedlings.

## Discussion

Recruitment of *E*. *fragilis* depended on the seed dispersal services provided by the endemic lizard *P*. *lilfordi*, which appears as its only disperser in the study island. Seed dispersal by lizards provided higher chances of success for both seeds and seedlings, with none of the monitored seedlings emerging from undispersed seeds surviving at the end of the study. This increased success provided by lizard dispersal was mediated by the pattern of seed distribution by lizards, while seed treatment in the digestive tract seems to have a negligible effect, as suggested by the similar emergence for seeds taken from red fruits and seeds digested by lizards. Seeds taken from lizard pellets were collected in the field, where red fruits are 4 times more frequent than yellow fruits. In addition, red fruits are preferred by lizards (12% higher consumption on red compared to yellow fruits in trials performed in the lab with 30 lizard individuals; CN pers. obs.). Therefore, we may assume that seeds in lizard pellets were mainly from red-fruited plants, thus the germination probability shown by digested seeds is probably the result of the effect of the colour, with a reduced effect of the digestion treatment.

Seed deposition patterns by dispersers link animal movement and behavior with spatial plant distribution and demography [39,40]. While undispersed seeds fall under their mother plants, seeds dispersed by lizards are moved to other microhabitats, reaching mainly open spaces and, in smaller proportions, *E*. *fragilis* or other shrubs. Under mother plants, seeds suffer higher predation and have lower probability of seedling emergence and survival. Lizards thus allow seeds to escape from a microhabitat where the probability of success is very limited.

The pattern of seed arrival to open sites is common for seeds dispersed by lizards (see also e.g. [10,30]). Lizards are ectotherms and need to spend more time in open areas to absorb sun radiation [41]. This pattern contrasts with that of frugivorous birds, which disperse most seeds to shrubs, where they usually spend most time perching (e.g. [42]). In Mediterranean ecosystems, characterized by a summer drought, recruitment is often facilitated beneath the canopy of other plants, where water loss is minimized [43]. However, *E*. *fragilis* recruitment was favoured in open sites, where c. 92.3% of recruitment occurred. This plant species is an early successional shrub [44] and is able to become established in open ground without a nurse object, which allows it to colonize new areas where seedling recruitment is often severely constrained [45]. Lizards are fundamental in allowing seeds to reach open sites where, once they become established, they can act as nurse plants for other species, accelerating succession towards more mature communities [46,47]. For this reason, *E*. *fragilis* has been suggested as an important keystone species for restoration programs in Mediterranean ecosystems [48,49]. Facilitation of the establishment of other species by pioneer nurse plants is especially important in arid and semiarid areas under Mediterranean climates where erosion is an important risk, and quick revegetation of empty sites is essential to stop and revert this trend [50,51].

The lizard *P*. *lilfordi* is an endangered species endemic to the Balearic Islands that is currently extinct from the biggest, most anthropized islands (Mallorca and Menorca), and has a decreasing population trend [28] in the small islands and islets where it still survives. Its extinction in the biggest islands has been related to habitat loss and the invasion of introduced carnivorous mammals [28]. The extinction of this lizard has caused the disruption of seed dispersal systems in these islands, having important consequences for population dynamics and genetics of lizard-dispersed plants ([52,53,54]; see also e.g. [55,56] for studies on the impact of the disruption of lizard-mediated dispersal on demography and genetics of plant populations in the Canary Islands). In the islands where it is still present, the Balearic lizard has a key role as pollinator and seed disperser (e.g. [20,30,57]). *Ephedra fragilis* is strongly dependent on *P*. *lilfordi*, which acted as its only disperser and had a clear positive effect on recruitment success of dispersed seeds compared to those undispersed. In addition, lizard preference for open sites makes them crucial for vegetation colonization and succession, especially in degraded lands. Land desertification in Mediterranean regions gives a key role to pioneer plant species, which acts as nurse plants and become important in the functioning of natural ecosystems as well as in restoration programs. But the population decline of the Balearic lizard threatens the maintenance of the ecological services it provides, especially when a strong dependency occurs, as that revealed in this study.

## Conclusions

Species diversity in natural communities as well as the generalism and diffuse nature that usually characterizes plant-animal interactions favours functional redundancy and a low interdependence between plant and animal partners, limiting the vulnerability of the community to co-extinctions and cascade effects. Strong dependencies in species interactions, in contrast, increase the vulnerability of species to the decline or eventual extinction of interacting partners. In the study system, recruitment of *E*. *fragilis* showed a strong dependency on the seed dispersal services provided by the endemic lizard *P*. *lilfordi*, which acts as its only disperser and increases the chances of success for both seeds and seedlings, with undispersed seeds failing to recruit in the study island. This strong dependency points to the vulnerability of *E*. *fragilis* to the extinction of its mutualist partner, and highlights the importance of conserving species interactions for biodiversity maintenance and ecosystem functioning.

## Supporting information

S1 Appendix Microhabitat cover and faeces distribution. Percentage cover of microhabitats and distribution of *Ephedra fragilis* seed dispersers’faeces.
(DOCX)

S1 Fig Microhabitat selection for seed dispersal by lizards. The percentage cover and the percentage of droppings found per each microhabitat are showed (mean ± SE). The difference between these percentages represent the selection for each particular microhabitat: equal percentages means no selection (the droppings deposited in this microhabitat are those expected by random seed dispersal among microhabitats), a higher percentage of droppings than of cover means a positive selection of this microhabitat for seed dispersal, and a lower percentage of droppings than of cover means a negative selection.
(TIF)
