# Supplementary material for: Strong dependence of a pioneer shrub on seed dispersal services provided by an endemic endangered lizard in a Mediterranean island ecosystem
Source: PLoS One. 2017 Aug 21;12(8):e0183072. doi: 10.1371/journal.pone.0183072 (PMC5565188; doi:10.1371/journal.pone.0183072)
Supplement: S1 Appendix — Percentage cover of microhabitats and distribution of Ephedra fragilis seed dispersers’faeces. (DOCX) [file pone.0183072.s001.docx]

**S1 Appendix. Percentage cover of microhabitats and distribution of *Ephedra fragilis* seed dispersers’faeces.**

Neghme, Santamaría and Calviño-Cancela. Strong dependence of a pioneer shrub on seed dispersal services provided by an endemic endangered lizard in a Mediterranean island ecosystem. PlosOne.

We established 3 transects 50 m long and 1 m wide running perpendicular to a path that crossed the study site in SW to NE direction and separates the two study zones (Zone A and B). We measured the surface covered by different plant species and open ground along the transects in order to estimate the percentage cover of each type of land cover. Most of the surface was open ground (45.1%), including rocks (14.4%) and bare soil (30.7%). *Pistacia lentiscus* was the most abundant shrub, covering 27.0% of the surface, followed by *E. fragilis* (17.5%), *Cneorum tricoccon* (3.6%) and *Phillyrea angustifolia* (3.1%), with other shrubs or herbs covering 3.5%. Along the same transects, we searched for faeces containing *E. fragilis* seeds within 1 m to each side of the transect, recording the type of microhabitat in which faeces were spotted. All faeces with *E. fragilis* seeds were of lizards. Although we found abundant faeces of small passerines, sea gulls, rats and rabbits, none contained *E. fragilis* seeds. Most lizard faeces were found in open areas (98.7%), with a small percentage being found under *E. fragilis* (0.71%) or *P. lentiscus* (0.71%) (Fig. S1). *Podarcis lilfordi*, the only frugivorous reptile in Dragonera, showed a positive selection of open areas for depositing their faeces, with the percentage of faeces arriving to this microhabitat being higher than that expected by a random distribution among microhabitats. In contrast, *E. fragilis* and *P. lentiscus* were negatively selected for dropping deposition (Fig. S1).

Fig. S1: Microhabitat selection for seed dispersal by lizards. The percentage cover and the percentage of droppings found per each microhabitat are showed (mean ± SE). The difference between these percentages represent the selection for each particular microhabitat: equal percentages means no selection (the droppings deposited in this microhabitat are those expected by random seed dispersal among microhabitats), a higher percentage of droppings than of cover means a positive selection of this microhabitat for seed dispersal, and a lower percentage of droppings than of cover means a negative selection.
