# Supplementary figures and images for: Strong dependence of a pioneer shrub on seed dispersal services provided by an endemic endangered lizard in a Mediterranean island ecosystem
Source: PLoS One. 2017 Aug 21;12(8):e0183072. doi: 10.1371/journal.pone.0183072 (PMC5565188; doi:10.1371/journal.pone.0183072)

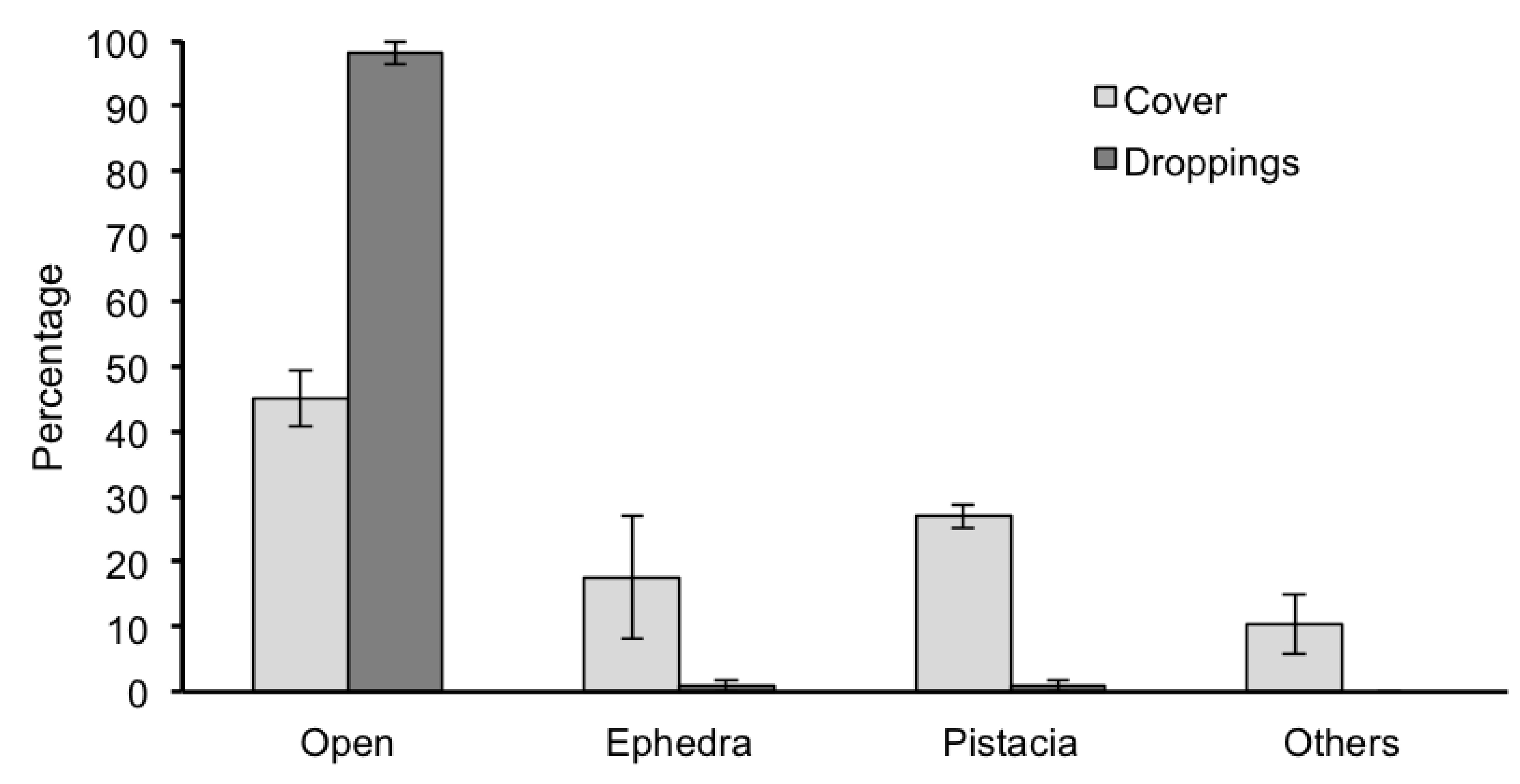

Supplement: S1 Fig — The percentage cover and the percentage of droppings found per each microhabitat are showed (mean ± SE). The difference between these percentages represent the selection for each particular microhabitat: equal percentages means no selection (the droppings deposited in this microhabitat are those expected by random seed dispersal among microhabitats), a higher percentage of droppings than of cover means a positive selection of this microhabitat for seed dispersal, and a lower percentage of droppings than of cover means a negative selection. (TIF) [file pone.0183072.s002.tif]
